# A Systematic Review and Meta-Analysis of Utility-Based Quality of Life in Chronic Kidney Disease Treatments

**DOI:** 10.1371/journal.pmed.1001307

**Published:** 2012-09-11

**Authors:** Melanie Wyld, Rachael Lisa Morton, Andrew Hayen, Kirsten Howard, Angela Claire Webster

**Affiliations:** 1 Sydney School of Public Health, University of Sydney, Sydney, New South Wales, Australia; 2 School of Public Health and Community Medicine, University of New South Wales, Sydney, New South Wales, Australia; 3 Centre for Transplant and Renal Research, Westmead Hospital, Westmead, New South Wales, Australia; University of Edinburgh, United Kingdom

## Abstract

**Background:**

Chronic kidney disease (CKD) is a common and costly condition to treat. Economic evaluations of health care often incorporate patient preferences for health outcomes using utilities. The objective of this study was to determine pooled utility-based quality of life (the numerical value attached to the strength of an individual's preference for a specific health outcome) by CKD treatment modality.

**Methods and Findings:**

We conducted a systematic review, meta-analysis, and meta-regression of peer-reviewed published articles and of PhD dissertations published through 1 December 2010 that reported utility-based quality of life (utility) for adults with late-stage CKD. Studies reporting utilities by proxy (e.g., reported by a patient's doctor or family member) were excluded.

In total, 190 studies reporting 326 utilities from over 56,000 patients were analysed. There were 25 utilities from pre-treatment CKD patients, 226 from dialysis patients (haemodialysis, *n* = 163; peritoneal dialysis, *n* = 44), 66 from kidney transplant patients, and three from patients treated with non-dialytic conservative care. Using time tradeoff as a referent instrument, kidney transplant recipients had a mean utility of 0.82 (95% CI: 0.74, 0.90). The mean utility was comparable in pre-treatment CKD patients (difference = −0.02; 95% CI: −0.09, 0.04), 0.11 lower in dialysis patients (95% CI: −0.15, −0.08), and 0.2 lower in conservative care patients (95% CI: −0.38, −0.01). Patients treated with automated peritoneal dialysis had a significantly higher mean utility (0.80) than those on continuous ambulatory peritoneal dialysis (0.72; *p* = 0.02). The mean utility of transplant patients increased over time, from 0.66 in the 1980s to 0.85 in the 2000s, an increase of 0.19 (95% CI: 0.11, 0.26). Utility varied by elicitation instrument, with standard gamble producing the highest estimates, and the SF-6D by Brazier et al., University of Sheffield, producing the lowest estimates. The main limitations of this study were that treatment assignments were not random, that only transplant had longitudinal data available, and that we calculated EuroQol Group EQ-5D scores from SF-36 and SF-12 health survey data, and therefore the algorithms may not reflect EQ-5D scores measured directly.

**Conclusions:**

For patients with late-stage CKD, treatment with dialysis is associated with a significant decrement in quality of life compared to treatment with kidney transplantation. These findings provide evidence-based utility estimates to inform economic evaluations of kidney therapies, useful for policy makers and in individual treatment discussions with CKD patients.

## Abstract 2

Melanie Wyld and colleagues examined previously published studies to assess pooled utility-based quality of life of the various treatments for chronic kidney disease. They conclude that the highest utility was for kidney transplants, with home-based automated peritoneal dialysis being second.

## Editors' Summary

**Background:**

Ill health can adversely affect an individual's quality of life, particularly if caused by long-term (chronic) conditions, such as chronic kidney disease—in the United States alone, 23 million people have chronic kidney disease, of whom 570,000 are treated with dialysis or kidney transplantation. In order to measure the cost-effectiveness of interventions to manage medical conditions, health economists use an objective measurement known as quality-adjusted life years. However, although useful, quality-adjusted life years are often criticized for not taking into account the views and preferences of the individuals with the medical conditions. A measurement called a utility solves this problem. Utilities are a numerical value (measured on a 0 to 1 scale, where 0 represents death and 1 represents full health) of the strength of an individual's preference for specified health-related outcomes, as measured by “instruments” (questionnaires) that rate direct comparisons or assess quality of life.

**Why Was This Study Done?:**

Previous studies have suggested that, in people with chronic kidney disease, quality of life (as measured by utility) is higher in those with a functioning kidney transplant than in those on dialysis. However, currently, it is unclear whether the type of dialysis affects quality of life: hemodialysis is a highly technical process that directly filters the blood, usually must be done 2–4 times a week, and can only be performed in a health facility; peritoneal dialysis, in which fluids are infused into the abdominal cavity, can be done nightly at home (automated peritoneal dialysis) or throughout the day (continuous ambulatory peritoneal dialysis). In this study, the researchers reviewed and assimilated all of the available evidence to investigate whether quality of life in people with chronic kidney disease (as measured by utility) differed according to treatment type.

**What Did the Researchers Do and Find?:**

The researchers did a comprehensive search of 11 databases to identify all relevant studies that included people with severe (stage 3, 4, or 5) chronic kidney disease, their form of treatment, and information on utilities—either reported directly, or included in quality of life instruments (SF-36), so the researchers could calculate utilities by using a validated algorithm. The researchers also recorded the prevalence rates of diabetes in study participants. Then, using statistical models that adjusted for various factors, including treatment type and the method of measuring utilities, the researchers were able to calculate the pooled utilities of each form of treatment for chronic kidney disease.

The researchers included 190 studies, representing over 56,000 patients and generating 326 utility estimates, in their analysis. The majority of utilities (77%) were derived through the SF-36 questionnaire via calculation. Of the 326 utility estimates, 25 were from patients pre-dialysis, 226 were from dialysis patients (the majority of whom were receiving hemodialysis), 66 were from kidney transplant patients, and three were from conservative care patients. The researchers found that the highest average utility was for those who had renal transplantation, 0.82, followed by the pre-dialysis group (0.80), dialysis patients (0.71), and, finally, patients receiving conservative care (0.62). When comparing the type of dialysis, the researchers found that there was little difference in utility between hemodialysis and peritoneal dialysis, but patients using automated peritoneal dialysis had, on average, a higher utility (0.80) than those treated with continuous ambulatory peritoneal dialysis (0.72). Finally, the researchers found that patient groups with diabetes had significantly lower utilities than those without diabetes.

**What Do These Findings Mean?:**

These findings suggest that in people with chronic kidney disease, renal transplantation is the best treatment option to improve quality of life. For those on dialysis, home-based automated peritoneal dialysis may improve quality of life more than the other forms of dialysis: this finding is important, as this type of dialysis is not as widely used as other forms and is also cheaper than hemodialysis. Furthermore, these findings suggest that patients who choose conservative care have significantly lower quality of life than patients treated with dialysis, a finding that warrants further investigation. Overall, in addition to helping to inform economic evaluations of treatment options, the information from this analysis can help guide clinicians caring for patients with chronic kidney disease in their discussions about possible treatment options.

**Additional Information:**

Please access these websites via the online version of this summary at http://dx.doi.org/10.1371/journal.pmed.1001307.

- Information about chronic kidney disease is available from the National Kidney Foundation and MedlinePlus
- Wikipedia gives information on general utilities (note that Wikipedia is a free online encyclopedia that anyone can edit; available in several languages)

## Introduction

Chronic kidney disease (CKD) is a common and costly condition to treat. In the United States in 2009, 7%–8% of the total population, around 23 million people, had CKD [1]. Of those, 570,000 were treated with dialysis or kidney transplantation [1]. In the United Kingdom there are an estimated 140,000 individuals with CKD under the care of a nephrologist [2], and an additional 50,000 who are treated with dialysis or kidney transplantation [3],[4]. In France, over 38,000 people in 16 of its 26 regions are treated with dialysis or kidney transplantation [4]. Similarly, in 12 of Italy's 20 regions almost 34,000 individuals rely on dialysis or kidney transplantation for survival [4]. CKD has an enormous impact on an individual's quality of life, and interventions like dialysis can influence this in either a positive or a negative direction. Quality of life estimates (utilities) are important for economic evaluations, as quality of life is a key component of economic benefit. Quality-adjusted life years are a measure of a person's length of life weighted by a valuation of their health-related quality of life over that period. Quality-adjusted life years are the preferred outcome in cost-effectiveness studies and enable direct comparisons to be made between treatment alternatives.

Utilities are the numerical value attached to the strength of an individual's preference for specific health-related outcomes. Utility is measured on a 0 to 1 scale, where 0 represents death and 1 represents full health [5]. It has been suggested that 0.03 is the minimum clinically important difference in utility [6], and this definition is applied in this study within the context of CKD. Utilities from single studies may not always be a reliable indicator of underlying quality of life, particularly where quality of life is not the main focus of the study but where quality of life data are collected as part of a broad set of study outcomes. Meta-analyses, on the other hand, have the advantage of combining all published data for a given population, potentially yielding more accurate utility estimates, as well as providing insight into the factors that influence quality of life.

From prior meta-analyses, it is known that utilities are lower in people with CKD than in those without kidney disease, and also that people with a functioning kidney transplant have higher utilities than people on dialysis [7],[8]. It is unclear how the type of dialysis impacts utility estimates, or how individuals who choose not to commence dialysis rate their quality of life. The need for greater understanding of outcomes for patients with end-stage kidney disease who forgo dialysis and are managed conservatively has been highlighted in the most recent annual report from the UK Renal Registry and in the US Renal Physicians Association's clinical practice guidelines on shared decision making in the appropriate initiation of, and withdrawal from, dialysis [3],[9].

Utility can be measured by a number of alternative approaches, using direct methods (such as time tradeoff and standard gamble) or multi-attribute utility instruments, such as the Australian Assessment of Quality of Life (http://www.aqol.com.au), the EuroQol Group's EQ-5D (http://www.euroqol.org), the UK's SF-6D (http://www.shef.ac.uk/scharr/sections/heds/mvh/sf-6d), the 15D from Finland (http://www.15d-instrument.net/15d), and the Health Utilities Index version 2 or 3 from Health Utilities (http://www.healthutilities.com) [5]. In addition, data from non-utility-based quality of life instruments such as the commonly used SF-36 health survey and the SF-12 health survey can be converted to a utility using published transformation algorithms [10],[11]. Using these algorithms allowed us to generate a more comprehensive meta-analysis than previously possible. The purpose of this study was to systematically review and determine pooled utility-based quality of life for CKD by treatment type.

## Methods

### Study Selection

This systematic review follows PRISMA guidelines (Text S1). We included all electronically available, peer-reviewed articles and PhD dissertations (herein referred to as studies) of any design. We included studies in languages other than English if they provided an English abstract. Abstracts for which a full study was not available (e.g., conference abstracts) were included if sufficient data for analysis were provided. No studies were excluded on the basis of sample size. Opinion pieces/editorials, meta-analyses, and systematic reviews were excluded. Studies were also excluded if they reported utilities from proxies (e.g., reported by a doctor or family member).

#### Participants

Studies were included if their sample population had stage 3, 4, or 5 CKD and were pre-dialysis, on a recognised form of kidney replacement therapy (haemodialysis, peritoneal dialysis, or kidney transplantation), or had chosen supportive non-dialytic therapy (also known as conservative care). Kidney disease staging was performed by each study and was not changed for our analysis. Pre-treatment CKD was defined as stage 3–5 CKD patients who did not yet require a form of kidney replacement therapy. All patients in included studies were 18 y of age or older. Studies of patients with acute kidney injury or who had received a combined pancreas-kidney transplant were excluded.

#### Utility-based quality of life

We included all studies that either reported utilities directly or where utilities could be calculated from SF-36 or SF-12 health surveys using a peer-reviewed algorithm [10],[11]. Studies that reported estimates from visual analogue scales, the Quality and Well-Being Scale, and the Rosser Index were excluded. Kidney Disease Quality of Life (KDQOL) scores were also excluded unless all eight SF-36 domains were reported separately and a utility could be calculated, as above.

### Search Methods

Using a specific renal search strategy based on one developed by the Cochrane Renal Group, and with input from the Cochrane Renal Group information management specialist, we searched 11 databases for articles published from database inception to 1 December 2010 (Text S2). MeSH terms and text words used are provided in Text S3. We undertook extensive searching of reference lists and conference proceedings and contacted relevant authors. This led to other unpublished grey literature such as PhD dissertations. Where there were multiple publications from the same study population, the most recent article that reported sufficient data for analysis was used unless there was a significant variation in sample size, in which case the study with the largest study population was used.

### Data Extraction and Management

Data from included studies were extracted onto a standardised data sheet by M. W. and R. L. M., with differences resolved through discussion. (Table S1). For non-English articles, native speakers were found to translate the articles where possible; otherwise, web-based translation tools were used. The reviewers were not blinded to study authors, affiliations, or journal name [12]. Variables recorded from each article included the following: publication year, number of patients, country, demographic and clinical characteristics of patients, type and time of treatment, and the utility estimates. We recorded the proportion of the study population with diabetes using prevalence rates or, if these were not reported, the rates for diabetic nephropathy from each sample.

In intervention studies, such as randomised controlled trials for new drugs, baseline characteristics were used to avoid the influence of the intervention on utility estimates. In studies where treatment groups were split by a clinical or demographic factor, the total group was used where possible. In longitudinal kidney transplant studies, utility at 12 mo was used, as this was considered a stable health state. In longitudinal studies of conservatively managed patients, baseline utility was used. Longitudinal analysis of utility-based quality of life was planned for all treatment groups (pre-treatment CKD, dialysis, conservative care, and transplantation).

### Data Analysis

#### Variance

When the standard deviation of a utility estimate was not reported, it was calculated, where possible, from the standard error. Because the standard deviation could not be calculated in many studies, we fitted a regression model using fractional polynomials of the observed standard deviations against utility estimates for those studies that provided a standard deviation [13].

#### Meta-regression

For the meta-regression, we fitted random effects models with robust estimation of standard errors to allow for potential clustering where studies provided more than one utility value [14]. This allowed us to use multiple utilities from a single study population. Because of missing data, we fitted separate models for each of the subgroups of interest (e.g., mean age group, year of publication), but adjusting for treatment modality (e.g., dialysis) and utility instrument in each model, where appropriate. We performed Wald tests to determine the significance of subgroups in the analyses.

## Results

### Study Characteristics

**Figure 1:**
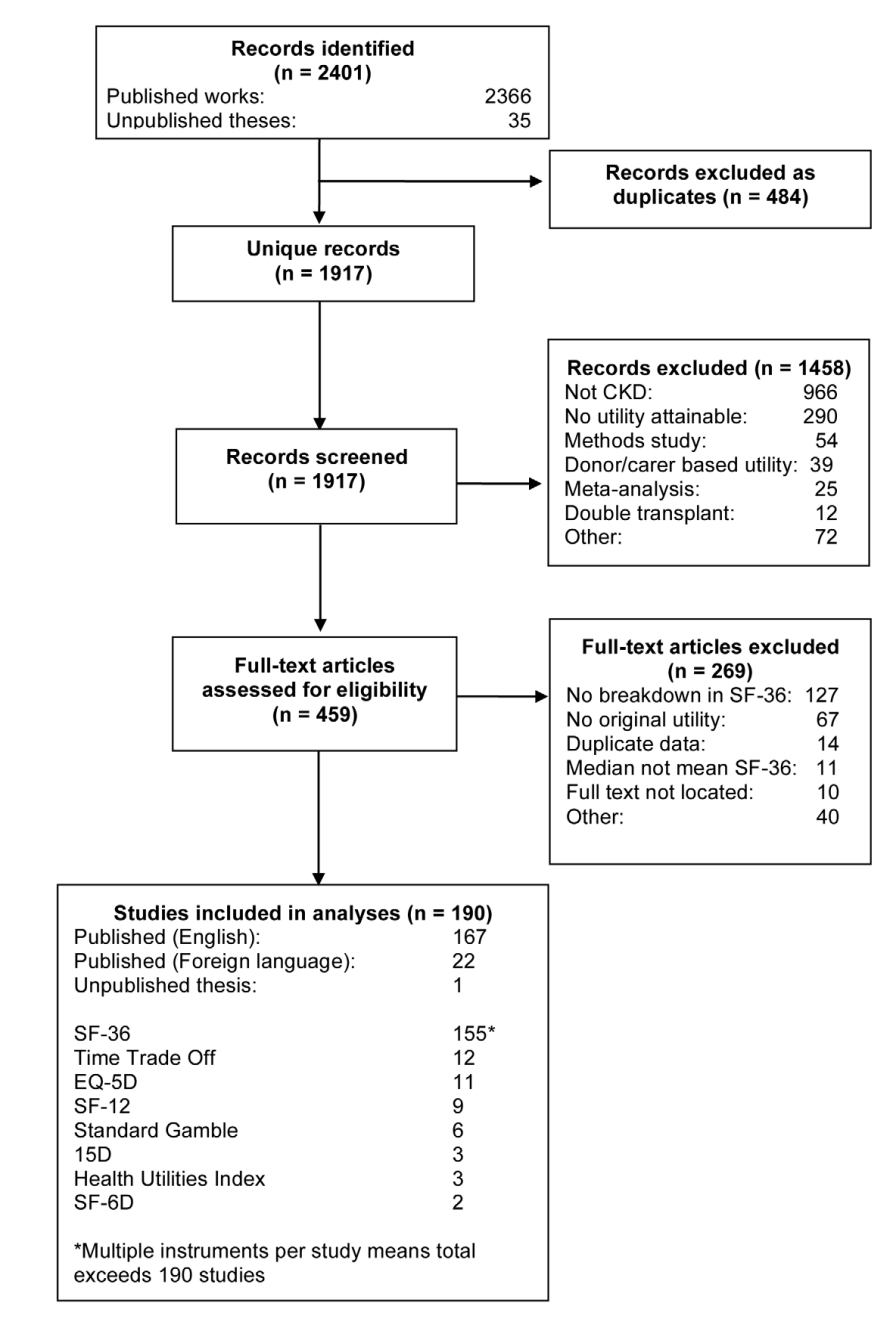
Flow diagram for derivation of studies included in the analyses.

The flow chart for identifying studies is shown in Figure 1. We included 190 studies representing over 56,000 patients (Text S4; Figure S1). The primary reason for exclusion was incomplete reporting of SF-36 domain scores (*n* = 127), which prohibited calculation of utility. Of the 190 studies, 22 (12%) were published in languages other than English. Ninety-two (48%) of the included studies reported more than one utility, generating a total of 326 utility estimates from the 190 studies.

Of the 326 utility estimates, 25 were from pre-treatment CKD patients, 226 were from dialysis patients, 66 were from kidney transplant patients, and three were from conservative care patients (Table 1). Six utilities were from patient populations receiving mixed treatments or where the treatment was unclear. The majority of utilities from dialysis patients were from patients treated with haemodialysis. The proportion of patients with diabetes was provided for 224 utilities (Table 1). The majority of utilities (*n* = 250, 77%) were derived through the SF-36 questionnaire (Table 1).

**Table 1:** Characteristics of included studies.

Category	Variable	Number of Utility Estimates	Percentage of Total Utility Estimates
**Treatment**	CKD (pre-treatment)	25	8%
	Dialysis (total)	226	69%
	Haemodialysis (total)	163	
	Home haemodialysis	6	
	In-center haemodialysis	153	
	Peritoneal dialysis (total)	44	
	Continuous ambulatory peritoneal dialysis	16	
	Automated peritoneal dialysis	6	
	Transplant	66	20%
	Conservative care	3	1%
	Mixed	6	2%
**Utility elicitation method**	Time tradeoff	31	10%
	Standard gamble	3	1%
	EQ-5D	23	7%
	EQ-5D derived from SF-12 health survey	10	3%
	EQ-5D derived from SF-36 health survey	250	77%
	15D	7	2%
	SF-6D	1	1%
**Geography**	US	99	30%
	Europe	151	46%
	Other	76	23%
**Diabetic rate**	0%	12	5%
	1%–33%	146	65%
	34%–66%	50	22%
	67%–99%	5	2%
	100%	11	5%
**Year of publication**	1980–1989	4	1%
	1990–1999	36	11%
	2000–2010	286	88%

Cross-sectional studies accounted for 216 (66%) utilities, cohort studies accounted for 57 (17%), case-control studies accounted for 34 (10%), and randomised controlled trials accounted for 16 (5%). Three estimates came from studies that were not one of those four study types. The majority of utility estimates had been published since the year 2000.

### Imputation of Standard Deviations

The standard deviation was available for 46 (14.1%) utility estimates. The utility estimates for which the standard deviation was available ranged from 0.39 to 0.94, and the utility estimates for which the standard deviation was missing ranged from 0.38 to 0.89. The equation for predicting the standard deviation of a utility estimate was standard deviation = 0.368−0.82×UtilityScore^2^+0.625×UtilityScore^3^.

### Utility Estimates by Kidney Disease Treatment Modality

The reference group in the model was kidney transplant patients with utility elicited via the time tradeoff instrument. The mean utility for this group was the highest, at 0.82 (95% CI: 0.74, 0.90), followed by the pre-treatment CKD group, 0.79 (95% CI: 0.70, 0.89), dialysis patients, 0.70 (95% CI: 0.62, 0.78), and conservative care patients, 0.62 (95% CI: 0.43, 0.82) (interaction *p*<0.001; Table 2).

**Table 2:** Model coefficient estimates, standard errors, and significance levels for predictors of utility-based quality of life.

Analysis	Factor	Coefficient Estimate (95% CI)	Standard Error	*p*-Value
**Treatment type and utility elicitation method**	**Intercept**	0.82 (0.74, 0.90)	0.04	<0.001
**Treatment effect (adjusted for utility elicitation method) (subgroup ** ***p*** **<0.001** a **)**	Transplant	0		
	CKD (pre-treatment)	−0.02 (−0.09, 0.04)	0.03	0.467
	Dialysis	−0.11 (−0.15, −0.08)	0.02	<0.001
	Conservative	−0.2 (−0.38, −0.01)	0.09	0.037
	Mixed	−0.06 (−0.12, 0.01)	0.03	0.089
**Utility elicitation method (adjusted for treatment effect) (subgroup ** ***p*** ** = 0.01)**	Time tradeoff	0		
	15D	0.05 (−0.10, 0.20)	0.07	0.53
	EQ-5D	−0.07 (−0.16, 0.01)	0.04	0.099
	EQ-5D derived from SF-12 health survey	−0.14 (−0.24, 0.04)	0.05	0.006
	EQ-5D derived from SF-36 health survey	−0.08 (−0.16, 0.00)	0.04	0.046
	SF-6D	−0.08 (−0.17, 0.00)	0.04	0.053
	Standard gamble	0.02 (−0.10, 0.14)	0.06	0.741
**Haemodialysis versus peritoneal dialysis (adjusted for utility elicitation method) (subgroup ** ***p*** ** = 0.075)**	**Intercept**	0.72	0.05	<0.001
	**Treatment effect**			
	Peritoneal dialysis	0		
	Haemodialysis	−0.03 (−0.06, 0.00)	0.02	0.075
**Automated peritoneal dialysis versus continuous ambulatory peritoneal dialysis (adjusted for utility elicitation method) (subgroup ** ***p*** ** = 0.021)**	**Intercept**	0.8 (0.69, 0.91)	0.06	<0.001
	**Treatment effect**			
	Automated peritoneal dialysis	0		
	Continuous ambulatory peritoneal dialysis	−0.08 (−0.14, −0.01)	0.03	0.021
**Diabetic status (adjusted for treatment type and utility elicitation method) (subgroup ** ***p*** **<0.001)**	**Intercept**	0.91 (0.82, 1.00)	0.05	<0.001
	**Diabetic rate**			
	0%	0		
	1%–33%	−0.04 (−0.07, −0.02)	0.01	0.002
	34%–66%	−0.07 (−0.10, −0.03)	0.02	<0.001
	67%–99%	−0.02 (−0.10, 0.06)	0.04	0.672
	100%	−0.11 (−0.17, −0.04)	0.03	0.001
**Transplantation utility by year of publication (adjusted for utility elicitation method) (subgroup ** ***p*** **<0.001)**	**Intercept**	0.66 (0.64, 0.69)	0.01	<0.001
	**Year of publication**			
	1980–1989	0		
	1990–1999	0.15 (0.06, 0.23)	0.04	<0.001
	2000–2010	0.19 (0.11, 0.26)	0.04	<0.001

a Wald tests were used to test the significance of subgroups.

There were 207 utility estimates specific to dialysis modality, either haemodialysis or peritoneal dialysis. While haemodialysis had a clinically lower mean utility estimate than peritoneal dialysis, 0.69 (95% CI: 0.59, 0.80) versus 0.72 (95% CI: 0.62, 0.83), the difference was not statistically significant (interaction *p* = 0.08; Table 2).

Within peritoneal dialysis treatment, still using the referent time tradeoff instrument, a significantly higher mean utility was found for patients treated with automated peritoneal dialysis (0.80; 95% CI: 0.69, 0.91) compared to those treated with continuous ambulatory peritoneal dialysis (0.72; 95% CI: 0.60, 0.85) (interaction *p* = 0.02; Table 2).

### Subgroup Analyses

#### Demographics

After adjusting for treatment type and utility instrument, mean patient age, which was available for 282 estimates, did not significantly influence utility (interaction *p* = 0.22). We were limited in our ability to further investigate the effect of age because of incomplete reporting and the use of mean age rather than patient-level data. Patient sex and geographic region also did not influence utility (interaction *p* = 0.37 and *p* = 0.07, respectively).

The percentage of patients with diabetes was reported for 224 utility estimates. There was a statistically significant difference in utility between patient groups with high, medium, or low rates of diabetes after adjusting for treatment type and utility instrument (interaction *p*<0.001). The group composed wholly of patients with diabetes had utilities 0.10 lower than those in the group without any diabetic patients, 0.81 (95% CI: 0.70, 0.91) versus 0.91 (95% CI: 0.82, 1.00) (Table 2).

#### Utility elicitation instrument and year of publication

Utility elicitation instrument was a statistically significant predictor of utility values (*p* = 0.01), with utilities converted from SF-36 and SF-12 questionnaires being significantly lower. EQ-5D estimates derived from the SF-36 were also generally lower than EQ-5D values acquired directly in studies where these two instruments were administered to the same patients (Table 3). There were no studies that administered both the SF-12 and EQ-5D instruments to the same patients. The year of publication was statistically significant for transplant utilities (interaction *p*<0.001): the mean utility estimate for kidney transplant was 0.66 between 1980 and 1989, 0.81 between 1990 and 1999, and 0.85 between 2000 and 2010, after controlling for elicitation instrument (Table 2).

**Table 3:** EQ-5D utility estimates reported directly and calculated from SF-36 for the same patient population.

Treatment	Study	Number of Patients	EQ-5D Direct Utility	EQ-5D Utility from SF-36	Difference
**Kidney transplant**	Lee et al. [16]	178	0.71	0.45	0.26 (37%)
**Haemodialysis**	Lee et al. [16]	99	0.44	0.30	0.14 (32%)
	Manns et al. [17]	128	0.60	0.47	0.13 (22%)
	Manns et al. [18]	151	0.62	0.48	0.14 (23%)
	Manns et al. [19], group 1	25	0.71	0.46	0.25 (35%)
	Manns et al. [19], group 2	26	0.58	0.49	0.19 (28%)
**Peritoneal dialysis**	Lee et al. [16]	74	0.53	0.33	0.20 (38%)
	Manns et al. [18]	41	0.56	0.47	0.09 (16%)

#### Longitudinal studies

Nine studies provided longitudinal data on mean utilities in the kidney transplant population (Table 4). Two studies reported longitudinal data for two different groups, resulting in longitudinal data for 11 patient groups. Of these 11 patient groups, only seven reported post-transplant utility over time. Two of these groups showed an increase in utility, and the remaining five groups showed no significant change. There were insufficient numbers of longitudinal studies in pre-treatment CKD, dialysis, and conservative management treatment groups to perform longitudinal analyses for these treatment groups.

**Table 4:** Longitudinal data for kidney transplant utility-based quality of life.

Study	Utility Elicitation Instrument	Number of Patients^a^	Utility				
			Pre-Transplant	Post-Transplant			
				0–3 mo	4–8 mo	9–12 mo	13–24 mo
Balaska et al. [20]	SF-36	85	0.35			0.60	
Laupacis et al. [21]	TTO	131	0.57	0.71	0.75	0.74	0.70
Oberbauer et al. [22], group 1	SF-36	183		0.61		0.62	0.62
Oberbauer et al. [22], group 2	SF-36	178		0.61		0.60	0.60
Painter et al. [23], group 1	SF-36	14		0.59		0.58	
Painter et al. [23], group 2	SF-36	9		0.67		0.69	
Perez San Gregorio et al. [24]	SF-36	28	0.59	0.57	0.63	0.64	
Pinson et al. [25]	SF-36	24	0.58	0.56			
Ravagnani et al. [26]	SF-36	17	0.57				0.61
Rodriguez et al. [27]	SF-36	31	0.56	0.57	0.62	0.65	
Russell et al. [28]	TTO	27	0.41			0.74	

a The populations varied over time in most studies. The minimum population reported for any time period was used.

TTO, time tradeoff instrument.

## Discussion

This meta-analysis has confirmed that mean utility is higher for kidney transplant patients than for dialysis patients [7],[8]. We have also found that utility is higher for transplant patients than for pre-treatment CKD patients. We have shown that patients on automated peritoneal dialysis have higher utility than patients on continuous ambulatory peritoneal dialysis, that diabetes has an adverse influence on utility for pre-treatment CKD patients, and that conservative care patients report the lowest utility of any treatment group. This work extends prior reviews in three ways: first, by analysing the effect of the era of publication on utility; second, by including a broader spectrum of patients, specifically those with pre-treatment CKD as well as conservative care patients; and, finally, by including all utility elicitation instruments, enabling comparisons across instruments to be made.

The finding that transplant patients' utilities increased significantly over time likely reflects improvements in transplant care and evolving clinical practice (e.g., the increasing number of immunosuppression drug options such as tacrolimus, mycophenolate mofetil, sirolimus, and steroid-free immunosuppression), and possibly a selection bias of younger patients with less co-morbidity accessing transplantation. It is possible that given the increased acceptance of higher risk patients into transplant programs over time, the utility effect of transplantation may be underestimated. The majority of longitudinal utility estimates for kidney transplant recipients did not show a clinically significant change. The small number of studies, with relatively short follow-up (2 y or less), suggests that this is an area that would benefit from additional research.

The type of utility elicitation instrument used was a statistically significant predictor of reported utility; some caution is therefore required when comparing values across instruments. The 15D, standard gamble, and time tradeoff instruments yielded utility estimates that were significantly higher than those from the EQ-5D (both directly measured as well as converted from the SF-36 and SF-12) and the SF-6D. We cannot recommend one particular instrument to be used in preference to others, as all instruments have their benefits and challenges in different settings. Instead, we offer criteria that may be considered in the choice of instrument. First, ensure that the instrument will measure the changes the study expects to see in the CKD population. For example, the EQ-5D may not be sensitive enough to detect changes in quality of life related to visual impairment (e.g., diabetic retinopathy) because it does not include a vision-specific domain. Second, review the instrument recommendations made by the study's funding bodies, e.g., the Pharmaceutical Benefits Advisory Committee in Australia, or the National Institute for Health and Clinical Excellence in the UK, the latter of which prefers the EQ-5D. Third, if utility-based quality of life is sought, a utility-based quality of life measure (e.g., time tradeoff or Health Utilities Index), rather than a generic questionnaire that is not utility based (e.g., SF-36 or SF-12), may be preferable. Recognise that if SF-36 or SF-12 is used, the derived utility will probably be lower than if utility has been measured directly. Finally, consider the frequency of the measurement. If repeated measurements are required, a simple instrument that can be completed quickly and reduces the likelihood of missing data may be preferable. There are other logistical issues that may also play a role in the decision to use one particular instrument (e.g., the availability of the preferred instrument in the local language). The use of these criteria should optimise the instrument choice for any particular purpose, setting, and clinical population.

There are a number of limitations to this study. First, treatment assignments were not random, limiting the strength of the conclusions that can be drawn from the findings. Second, we could not adequately account for demographic differences such as age and sex, nor for clinical differences such as delivered dialysis dose or kidney transplant function, because of the incompleteness of reported data and our reliance on the aggregated, rather than patient-level, data provided by studies. The form of the aggregated data we assembled meant it was not possible to perform an additional meta-regression to assess whether differences in the study characteristics accounted for heterogeneity. Third, we calculated EQ-5D scores from SF-36 and SF-12 data, and the algorithms may not reflect what the EQ-5D scores would have been had they been measured directly. This is of particular note given the large number of studies that used SF-36 data. Additionally, because these EQ-5D scores were calculated, we had to impute standard deviations, and this may have affected the results. Fourth, there was an insufficient number of studies of home haemodialysis to conduct a separate analysis for this sub-modality. Fifth, there were just three studies that explored the utility of conservative care patients, limiting the conclusions that can be drawn about the quality of life of patients who choose to forgo dialysis. Sixth, longitudinal data were available only for the transplant population. Finally, our search was conducted in December 2010, and additional relevant studies may have been published since then.

The strengths of this review are its size and comprehensiveness. By including 326 utilities from over 56,000 participants, this analysis is substantially larger than previous reviews [7],[8]. The size of this review enabled subgroup analyses that revealed previously unknown findings. Additionally, this review was comprehensive as it included non-English-language articles and unpublished theses as well as a broader patient population, including pre-treatment CKD and conservative care patients, groups that have not to our knowledge been included in previous analyses.

### Implications for Clinical Practice

Our results suggest that automated peritoneal dialysis, a home-based form of dialysis that accounts for just 7% of dialysis patients in the UK and 4% of dialysis patients in the United States [1],[3], has a significantly higher mean utility than continuous ambulatory peritoneal dialysis. We interpret this finding with caution, as these utility estimates were not drawn from randomised comparisons, and patient groups treated with different modalities are likely to be different (e.g., differences in burden of co-morbidities or degree of family support). However, our findings do suggest that an expansion of automated peritoneal dialysis in clinical practice may be appropriate where possible. The pooled utility estimates for the two types of dialysis may inform discussions with patients about the benefits and harms of different dialysis sub-modalities, particularly in cases where there is uncertainty or equipoise in terms of modality-specific survival.

Areas for further research include longitudinal assessment of kidney transplant and dialysis patients' utility-based quality of life, the accuracy of the algorithm that translates SF-36 scores into EQ-5D scores, and the quality of life experienced by patients who choose conservative care. Further research assessing quality of life with home-based dialysis modalities (i.e., home haemodialysis and peritoneal dialysis) would help determine to what extent the higher utilities seen for automated peritoneal dialysis reflect the location of treatment, rather than the type of treatment.

### Conclusions

This research is to our knowledge the largest meta-analysis on this topic to date, and includes eight different utility instruments, with published as well as unpublished studies from English- and non-English-language journals. Within the dialysis population, the highest utility of the sub-modalities was reported by those on home-based automated peritoneal dialysis. This finding suggests that the management of patients on automated peritoneal dialysis is beneficial in CKD care. This study has also shown that utility-based quality of life for transplant recipients has been improving over time, with clear increases in mean utility since the 1980s. We found that patients who chose conservative care had significantly lower quality of life than patients treated with dialysis, an area that requires further research. These findings can be used in economic evaluations of kidney therapies, and may also be useful in treatment discussions with patients.

## Supporting Information

Table S1 **Study details for the 326 utilities.**
(XLS)

Text S1 **PRISMA checklist.**
(DOC)

Text S2 **Databases searched.**
(DOCX)

Text S3 **Summary of terms used in the Medline search strategy.**
(DOCX)

Text S4 **References for studies included in the meta-analysis.**
(DOCX)

## Abbreviations

CKD: chronic kidney disease
