# Supplementary material for: A Systematic Review and Meta-Analysis of Utility-Based Quality of Life in Chronic Kidney Disease Treatments
Source: PLoS Med. 2012 Sep 11;9(9):e1001307. doi: 10.1371/journal.pmed.1001307 (PMC3439392; doi:10.1371/journal.pmed.1001307)
Supplement: Text S2 — Databases searched. (DOCX) [file pmed.1001307.s003.docx]

**Text S2 Databases searched.**

Australian Digital Theses

British Library Electronic Digital Thesis Online Service (EThOS)

Cinahl

Cochrane Database of Systematic Review

EconLit

Embase

Health Technology Assessment

Medline

NHS Economic Evaluation Database

PreMedline

ProQuest Dissertation and Theses

PsychInfo (1987-2009)

Scopus

Thesis Canada Portal

Tufts Cost Effectiveness Analysis Register
