# Supplementary figures and images for: A Systematic Review and Meta-Analysis of Utility-Based Quality of Life in Chronic Kidney Disease Treatments
Source: PLoS Med. 2012 Sep 11;9(9):e1001307. doi: 10.1371/journal.pmed.1001307 (PMC3439392; doi:10.1371/journal.pmed.1001307)

**Text S3 Summary of terms used in the Medline search strategy.**


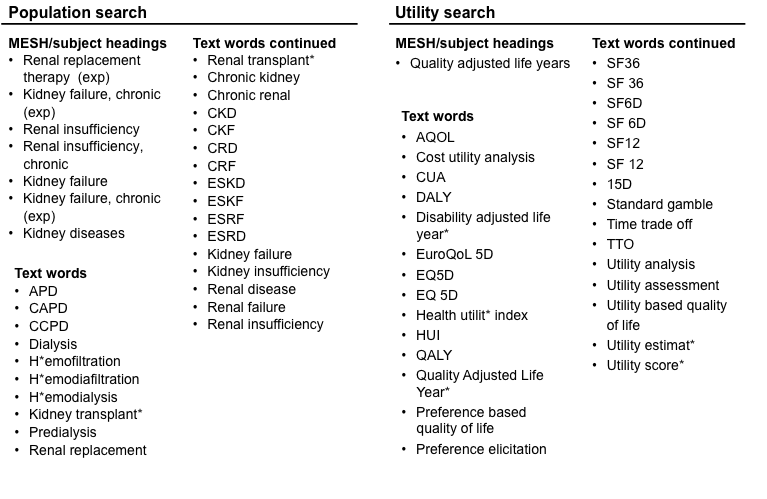

Supplement: Text S3 — Summary of terms used in the Medline search strategy. (DOCX) [file pmed.1001307.s004.docx]
