# Supplementary material for: A Systematic Review and Meta-Analysis of Utility-Based Quality of Life in Chronic Kidney Disease Treatments
Source: PLoS Med. 2012 Sep 11;9(9):e1001307. doi: 10.1371/journal.pmed.1001307 (PMC3439392; doi:10.1371/journal.pmed.1001307)
Supplement: Text S4 — References for studies included in the meta-analysis. (DOCX) [file pmed.1001307.s005.docx]

**Text S4 References for studies included in the meta-analysis.**

1. Aasebo W, Homb-Vesteraas NA, Hartmann A, Stavem K. Life situation and quality of life in young adult kidney transplant recipients. Nephrol Dial Transplant. 2009 Jan;24(1):304-8.
2. Aasebo W, Midtvedt K, Hartmann A, Stavem K. Predictors of health-related quality of life in hypertensive recipients following renal transplantation. Clin Transplant. 2005 Dec;19(6):756-62.
3. Acaray A, Pinar R. Quality of life in Turkish haemodialysis patients. Int Urol Nephrol. 2005;37(3):595-602.
4. Afsar B, Elsurer R, Eyileten T, Yilmaz MI, Caglar K. Antibody response following hepatitis B vaccination in dialysis patients: does depression and life quality matter? Vaccine. 2009 Sep 25;27(42):5865-9.
5. Alexander M, Kewalramani R, Agodoa I, Globe D. Association of anemia correction with health related quality of life in patients not on dialysis. Curr Med Res Opin. 2007 Dec;23(12):2997-3008.
6. Alfaro Cuenca A, Beltrán Redondo I, Gallego Jordán B, Martín Piñero M, Romero Espinosa E, et al. In line HDF in our patients: quality of life and functional capacity. [Spanish] Revista de la Sociedad Española de Enfermería Nefrológica. 2006;9(3):6-11.
7. Altintepe L, Levendoglu F, Okudan N, Guney I, Savas Cilli A, et al. Physical disability, psychological status, and health-related quality of life in older hemodialysis patients and age-matched controls. Hemodial. 2006 Jul;10(3):260-6.
8. Apostolou T, Hutchison AJ, Boulton AJM, Chak W, Vileikyte L, et al. Quality of life in CAPD, transplant, and chronic renal failure patients with diabetes. Ren Fail. 2007;29(2):189-97.
9. Ardine de Wit G, Ramsteijn PG, de Charro FT. Economic evaluation of end stage renal disease treatment. 1998;44:215-32.
10. Arenas VG, Barros LFN, Lemos FB, Martins MA, David-Neto E. Quality of life: comparison between patients on automated peritoneal dialysis and patients on hemodialysis [Portuguese]. Acta Paulista de Enfermagem. 2009;22:535-9.
11. Arogundade FA, Zayed B, Daba M, Barsoum RS. Correlation between Karnofsky Performance Status Scale and Short-Form Health Survey in patients on maintenance hemodialysis. J Natl Med Assoc. 2004 Dec;96(12):1661-7.
12. Baiardi F, Degli Esposti E, Cocchi R, Fabbri A, Sturani A, et al. Effects of clinical and individual variables on quality of life in chronic renal failure patients. Jn, J. 2002 Jan-Feb;15(1):61-7.
13. Balaska A, Moustafellos P, Gourgiotis S, Pistolas D, Hadjiyannakis E, et al. Changes in health-related quality of life in Greek adult patients 1 year after successful renal transplantation. Exp Clin Transplant. 2006 Dec;4(2):521-4.
14. Basok EK, Atsu N, Rifaioglu MM, Kantarci G, Yildirim A, et al. Assessment of female sexual function and quality of life in predialysis, peritoneal dialysis, hemodialysis, and renal transplant patients. Int Urol Nephrol. 2009;41(3):473-81.
15. Bass EB, Wills S, Fink NE, Jenckes MW, Sadler JH, et al. How strong are patients' preferences in choices between dialysis modalities and doses? Am J Kidney Dis. 2004 Oct;44(4):695-705.
16. Beretta E, Di Mauro S, Galimberti S. Quality of life of recently dialysed patients: the comparison of two pre-dialysis interventions [Italian]. Assist Inferm Ric. 2009 Apr-Jun;28(2):82-8.
17. Bilgic A, Akman B, Sezer S, Ozisik L, Arat Z, et al. Predictors for quality of life in continuous ambulatory peritoneal dialysis patients. Nephrology. 2008 Oct;13(7):587-92.
18. Bohlke M, Marini SS, Rocha M, Terhorst L, Gomes RH, et al. Factors associated with health-related quality of life after successful kidney transplantation: A population-based study. Quality of Life Research: An International Journal of Quality of Life Aspects of Treatment, Care & Rehabilitation. 2009 Nov;18(9):1185-93.
19. Bohlke M, Nunes DL, Marini SS, Kitamura C, Andrade M, et al. Predictors of quality of life among patients on dialysis in southern Brazil. Sao Paulo Med J. 2008 Sep;126(5):252-6.
20. Bossola M, Giungi S, Luciani G, Tazza L. Body mass index, comorbid conditions and quality of life in hemodialysis patients. Jn, J. 2009 Jul-Aug;22(4):508-14.
21. Buemi M, Caccamo C, Floccari F, Coppolino G, Tripodo D, et al. Correlation between quality of life assessment and a personality neurobiologic model in dialyzed patients. Jn, J. 2003 Nov-Dec;16(6):895-902.
22. Cafazzo JA, Leonard K, Easty AC, Rossos PG, Chan CT. Patient-perceived barriers to the adoption of nocturnal home hemodialysis.[see comment]. Clin J Am Soc Nephrol. 2009 Apr;4(4):784-9.
23. Canadian Erythropoietin Study Group, Association between recombinant human erythropoietin and quality of life and exercise capacity of patients receiving haemodialysis. BMJ. 1990 Mar 3;300(6724):573-8.
24. Carmichael P, Popoola J, John I, Stevens PE, Carmichael AR. Assessment of quality of life in a single centre dialysis population using the KDQOL-SF questionnaire. Qual Life Res. 2000 Mar;9(2):195-205.
25. Caskey FJ, Wordsworth S, Ben T, de Charro FT, Delcroix C, et al. Early referral and planned initiation of dialysis: what impact on quality of life? Nephrol Dial Transplant. 2003 Jul;18(7):1330-8.
26. Chang ST, Chen CL, Chen CC, Lin FC, Wu D. Enhancement of quality of life with adjustment of dry weight by echocardiographic measurement of inferior vena cava diameter in patients undergoing chronic hemodialysis. Nephron [serial on the Internet]. 2004; (3): Available from: [http://www.mrw.interscience.wiley.com/ cochrane/clcentral/articles/323/CN-00520323/frame.html](http://www.mrw.interscience.wiley.com/%20cochrane/clcentral/articles/323/CN-00520323/frame.html).
27. Chen YC, Hung KY, Kao TW, Tsai TJ, Chen WY. Relationship between dialysis adequacy and quality of life in long-term peritoneal dialysis patients. Perit Dial Int. 2000 Sep-Oct;20(5):534-40.
28. Chiang CK, Peng Y-S, Chiang S-S, Yang C-S, He Y-H, et al. Health-related quality of life of hemodialysis patients in Taiwan: a multicenter study. Blood Purif. 2004;22(6):490-8.
29. Chen CK, Tsai YC, Hsu HJ, Wu IW, Sun CY, et al. Depression and suicide risk in hemodialysis patients with chronic renal failure. Psychosomatics. 2010;51(6):528-.e6.
30. Chisholm MA, Spivey CA, Nus AV. Influence of economic and demographic factors on quality of life in renal transplant recipients. Clin Transplant. 2007 Mar-Apr;21(2):285-93.
31. Chow FYF, Briganti EM, Kerr PG, Chadban SJ, Zimmet PZ, et al. Health-related quality of life in Australian adults with renal insufficiency: a population-based study. Am J Kidney Dis. 2003 Mar;41(3):596-604.
32. Chow KM, Szeto CC, Kum LC-C, Kwan BCH, Fung TM-K, et al. Improved health-related quality of life and left ventricular hypertrophy among dialysis patients treated with parathyroidectomy. Jn, J. 2003 Nov-Dec;16(6):878-85.
33. Cleary J, Drennan J. Quality of life of patients on haemodialysis for end-stage renal disease. J Adv Nurs. 2005;51(6):577-86.
34. Cleemput I, Kesteloot K, Moons P, Vanrenterghem Y, Van Hooff JP, et al. The construct and concurrent validity of the EQ-5D in a renal transplant population. Value Health. 2004 Jul-Aug;7(4):499-509.
35. Cleemput I, Kesteloot K, De Geest S, Dobbels F, Vanrenterghem Y. Health professionals' perceptions of health status after renal transplantation: a comparison with transplantation candidates' expectations. Transplantation. 2003 Jul 15;76(1):176-82.
36. Churchill DN, Torrance GW, Taylor DW, Barnes CC, Ludwin D, et al. Measurement of quality of life in end-stage renal disease: the time trade-off approach. Clin Invest Med. 1987 Jan;10(1):14-20.
37. Contreras F, Esguerra GA, Espinosa JC, Gomez V. Coping styles and quality of life in patients with chronic kidney disease (CKD) in treatment with haemodialysis [Spanish]. Acta Colombiana de Psicologia. 2007 Jul-Dec;10(2):169-79.
38. Cruz LN, Fleck MPdA, Polanczyk CA. Depression as a determinant of quality of life in patients with chronic disease: data from Brazil. Soc Psychiatry Psychiatr Epidemiol. 2010 Oct;45(10):953-61.
39. Culleton BF, Walsh M, Klarenbach SW, Mortis G, Scott-Douglas N, et al. Effect of frequent nocturnal hemodialysis vs conventional hemodialysis on left ventricular mass and quality of life: A randomized controlled trial. JAMA: Journal of the American Medical Association. 2007 Sep;298(11):1291-9.
40. Davidson SN, Jhangri GS. Comparing the Health Utilities Index Mark 3 (HUI3) with the Short Form-36 Preference Based SF-6D in Chronic Kidney Disease. Value Health. 2009;12(2):340-5.
41. Davison SN, Jhangri GS. Existential and religious dimensions of spirituality and their relationship with health-related quality of life in chronic kidney disease. Clinical Journal of the American Society of Nephrology. 2010;5(11):1969-76.
42. Davidson SN, Jhangri GS. Impact of Pain and Symptom Burden on the Health-Related Quality of Life of Hemodialysis Patients. Journal of Pain and Symptom Management. 2010;39(3):477-85.
43. de Castro M, Caiuby AVS, Draibe SA, Canziani MEF. Quality of life in chronic renal disease patients submitted to hemodialysis evaluated with SF-36 instrument [Portuguese]. Rev Assoc Med Bras. 2003 Jul-Sep;49(3):245-9.
44. De Moura Reboredo M, Henrique DMN, De Souza Faria R, Chaoubah A, Bastos MG, et al. Exercise training during hemodialysis reduces blood pressure and increases physical functioning and quality of life. Artif Organs. 2010;34(7):586-93.
45. DeOreo PB. Hemodialysis patient-assessed functional health status predicts continued survival, hospitalization, and dialysis-attendance compliance. Am J Kidney Dis. 1997 Aug;30(2):204-12.
46. de Wit GA, Merkus MP, Krediet RT, de Charro FT. Health profiles and health preferences of dialysis patients. Nephrol Dial Transplant. 2002 Jan;17(1):86-92.
47. de Wit GA, Merkus MP, Krediet RT, de Charro FT. A comparison of quality of life of patients on automated and continuous ambulatory peritoneal dialysis. Perit Dial Int. 2001 May-Jun;21(3):306-12.
48. Diamant MJ, Harwood L, Movva S, Wilson B, Stitt L, et al. A comparison of quality of life and travel-related factors between in-center and satellite-based hemodialysis patients. Clin J Am Soc Nephrol. 2010 Feb;5(2):268-74.
49. Diaz-Buxo JA, Lowrie EG, Lew NL, Zhang H, Lazarus JM. Quality-of-life evaluation using Short Form 36: comparison in hemodialysis and peritoneal dialysis patients. Am J Kidney Dis. 2000 Feb;35(2):293-300.
50. Diaz-Dominguez R, Perez-Bernal J, Perez-San-Gregorio MA, Martin-Rodriguez A. Quality of life in patients with kidney, liver or heart failure during the waiting list period. Transplant Proc. 2006 Oct;38(8):2459-61.
51. Fassett RG, Robertson IK, Geraghty DP, Ball MJ, Burton NW, et al. Physical activity levels in patients with chronic kidney disease entering the LORD trial. Med Sci Sports Exerc. 2009 May;41(5):985-91.
52. Finkelstein FO, Story K, Firanek C, Mendelssohn D, Barre P, et al. Health-related quality of life and hemoglobin levels in chronic kidney disease patients. Clin J Am Soc Nephrol. 2009 Jan;4(1):33-8.173.
53. Fitzsimons D, Mullan D, Wilson JS, Conway B, Corcoran B, et al. The challenge of patients' unmet palliative care needs in the final stages of chronic illness. Palliat Med. 2007;21(4):313-22.
54. Frank A, Auslander GK, Weissgarten J. Quality of life of patients with end-stage renal disease at various stages of the illness. Soc Work Health Care. 2003;38(2):1-27.
55. Fujisawa M, Ichikawa Y, Yoshiya K, Isotani S, Higuchi A, et al. Assessment of health-related quality of life in renal transplant and hemodialysis patients using the SF-36 health survey. Urology. 2000 Aug 1;56(2):201-6.
56. Fukuhara S, Akizawa T, Morita S, Koshikawa S, Group KAS. Quality of life improvements in dialysis patients receiving darbepoetin alfa. Therap Apher Dial. 2008 Feb;12(1):72-7.
57. Fukuhara S, Yamazaki S, Marumo F, Akiba T, Akizawa T, et al. Health-related quality of life of predialysis patients with chronic renal failure. Nephron. 2007;105(1):c1-8.
58. Gataa R, Ajmi TN, Haouala F, Mtiraoui A. Quality of life patterns of dialysed patients in the region of Kairouan [French]. Tunis Med. 2008 Jan;86(1):68-74.
59. Gentile S, Boini S, Germain L, Jacquelinet C, Bloch J, et al. Quality of life of dialysis and renal transplant patients: Results of two multiregional surveys, France [French]. Bulletin Epidemiologique Hebdomadaire. 2010;9-10:92-6.
60. Goetzmann L, Sarac N, Ambuhl P, Boehler A, Irani S, et al. Psychological response and quality of life after transplantation: a comparison between heart, lung, liver and kidney recipients. Swiss Med Wkly. 2008 Aug 23;138(33-34):477-83.
61. Gorodetskaya I, Zenios S, McCulloch CE, Bostrom A, Hsu CY, et al. Health-related quality of life and estimates of utility in chronic kidney disease. Kidney Int. 2005;68(6):2801-8.
62. Goma AS, Peris PA, Alcario ABR. Quality of life of patients with chronic kidney disease undergoing renal with dialysis therapy [Spanish]. Calidad de vida en pacientes con insuficiencia renal cronica en tratamiento con dialisis. 2010;13(3):155-60.
63. Griva K, Ziegelmann JP, Thompson D, Jayasena D, Davenport A, et al. Quality of life and emotional responses in cadaver and living related renal transplant recipients. Nephrol Dial Transplant. 2002 Dec;17(12):2204-11.
64. Guerini Rocco D, Mercieri A, Yavuzer G. Multidimensional health-status assessment of chronic hemodialysis patients: the impact on quality of life. Eur. 2006 Jun;42(2):113-9.
65. Gumprecht J, Zelobowska K, Gosek K, Zywiec J, Adamski M, et al. Quality of life among diabetic and non-diabetic patients on maintenance haemodialysis. Exp Clin Endocrinol Diabetes. 2010 Mar;118(3):205-8.
66. Hallinen T, Soini EJO, Martikainen JA, Ikaheimo R, Ryynanen O-P. Costs and quality of life effects of the first year of renal replacement therapy in one Finnish treatment centre. J Med Econ. 2009;12(2):136-40.
67. Hamilton G, Locking-Cusolito H. Hemodialysis adequacy and quality of life: how do they relate? J Cannt. 2003 Oct-Dec;13(4):24-9.
68. Han SS, Kim KW, Na KY, Chae D-W, Kim YS, et al. Quality of life and mortality from a nephrologist's view: a prospective observational study. BMC Nephrol. 2009;10:39.
69. He Q, Chen J, Xu Y, Zhang P, Xie W, et al. High-risk end-stage renal disease patients converted from conventional to short daily haemodialysis. J Int Med Res. 2006 Nov-Dec;34(6):682-8.
70. Houle N, Bohannon RW, Frigon L, Maljanian R, Nieszczezewski J. Health promoting behaviors, quality of life, and hospital resource utilization of patients receiving kidney transplants. Nephrology Nursing Journal. 2002;29(1):35.
71. Iliescu EA, Coo H, McMurray MH, Meers CL, Quinn MM, et al. Quality of sleep and health-related quality of life in haemodialysis patients.[see comment]. Nephrol Dial Transplant. 2003 Jan;18(1):126-32.
72. Jassal SV, Brissenden JE, Raisbeck A, Roscoe JM. Comparative cost-analysis of two different chronic care facilities for end-stage renal disease patients. Geriatr Nephrol Urol. 1998;8(2):69-76.
73. Jiang M-m, Li L. Assessment of health-related quality of life in hemodialysis patients with SF-36 [Chinese]. Zhejiang Da Xue Xue Bao Yi Xue Ban. 2004 Nov;33(6):546-9.
74. Juergensen PH, Zemchenkov A, Watnick S, Finkelstein S, Wuerth D, et al. Comparison of quality-of-life assessment in Russia and the United States in chronic peritoneal dialysis patients. Adv Perit Dial. 2002;18:55-7.
75. Kalantar-Zadeh K, Kopple JD, Kamranpour N, Fogelman AM, Navab M. HDL-inflammatory index correlates with poor outcome in hemodialysis patients. Kidney Int. 2007 Nov;72(9):1149-56.
76. Kalantar-Zadeh K, Kopple JD, Block G, Humphreys MH. Association among SF36 quality of life measures and nutrition, hospitalization, and mortality in hemodialysis. J Am Soc Nephrol. 2001 Dec;12(12):2797-806.
77. Kalender B, Ozdemir AC, Dervisoglu E, Ozdemir O. Quality of life in chronic kidney disease: effects of treatment modality, depression, malnutrition and inflammation. Int J Clin Pract. 2007 Apr;61(4):569-76.
78. Kawada N, Moriyama T, Ichimaru N, Imamura R, Matsui I, et al. Negative effects of anemia on quality of life and its improvement by complete correction of anemia by administration of recombinant human erythropoietin in posttransplant patients. Clin Exp Nephrol. 2009 Aug;13(4):355-60.
79. Kawauchi A, Inoue Y, Hashimoto T, Tachibana N, Shirakawa S, et al. Restless legs syndrome in hemodialysis patients: health-related quality of life and laboratory data analysis. Clin Nephrol. 2006 Dec;66(6):440-6.
80. Khan IH, Garratt AM, Kumar A, Cody DJ, Catto GR, et al. Patients' perception of health on renal replacement therapy: evaluation using a new instrument. Nephrol Dial Transplant. 1995;10(5):684-9.
81. Kim K-S, Kang J-Y, Jeong I-S. Health related quality of life among organ transplant recipients [Korean]. Taehan Kanho Hakhoe Chi. 2003 Jun;33(3):365-75.
82. Kontodimopoulos N, Pappa E, Niakas D. Gender- and age-related benefit of renal replacement therapy on health-related quality of life. Scandinavian Journal of Caring Sciences. 2009;23(4):721-9.
83. Kurtin PS, Davies AR, Meyer KB, DeGiacomo JM, et al. Patient-based health status measures in outpatient dialysis: Early experiences in developing an outcomes assessment program. Med Care. 1992 May;30(5, Suppl):136-49.
84. Kusek JW, Greene P, Wang S-R, Beck G, West D, et al. Cross-sectional study of health-related quality of life in African Americans with chronic renal insufficiency: the African American Study of Kidney Disease and Hypertension Trial. Am J Kidney Dis. 2002 Mar;39(3):513-24.
85. Kusleikaite N, Bumblyte IA, Pakalnyte R. Quality of life and depression in renal transplant patients [Lithuanian]. Medicina (Kaunas). 2007;43 Suppl 1:103-8.
86. Kusleikaite N, Bumblyte IA, Kuzminskis V, Vaiciuniene R. The association between health-related quality of life and mortality among hemodialysis patients [Lithuanian]. Medicina (Kaunas). 2010;46(8):531-7.
87. Kusleikaite N, Bumblyte IA, Razukeviciene L, Sedlickaite D, Rinkunas K. Sleep disorders and quality of life in patients on hemodialysis [Lithuanian]. Medicina (Kaunas). 2005;41 Suppl 1:69-74.
88. Kutner NG, Zhang R, McClellan WM. Patient-reported quality of life early in dialysis treatment: effects associated with usual exercise activity... including commentary by Hoffart N with author response. Nephrology Nursing Journal. 2000;27(4):357.
89. Lacson E, Jr., Xu J, Lin S-F, Dean SG, Lazarus JM, et al. A comparison of SF-36 and SF-12 composite scores and subsequent hospitalization and mortality risks in long-term dialysis patients. Clin J Am Soc Nephrol. 2010 Feb;5(2):252-60.
90. Laupacis A, Keown P, Pus N, Krueger H, Ferguson B, et al. A study of the quality of life and cost-utility of renal transplantation. Kidney Int. 1996 Jul;50(1):235-42.
91. Lausevic M, Nesic V, Stojanovic M, Stefanovic V. Health-related quality of life in patients on peritoneal dialysis in Serbia: comparison with hemodialysis. Artif Organs. 2007 Dec;31(12):901-10.
92. Lee AJ, Morgan CL, Conway P, Currie CJ. Characterisation and comparison of health-related quality of life for patients with renal failure. Curr Med Res Opin. 2005 Nov;21(11):1777-83.
93. Lee S-Y, Lee H-J, Kim Y-K, Kim S-H, Kim L, et al. Neurocognitive function and quality of life in relation to hematocrit levels in chronic hemodialysis patients. J Psychosom Res. 2004 Jul;57(1):5-10.
94. Levendoglu F, Altintepe L, Okudan N, Ugurlu H, Gokbel H, et al. A twelve week exercise program improves the psychological status, quality of life and work capacity in hemodialysis patients. Jn, J. 2004 Nov-Dec;17(6):826-32.
95. Lew-Starowicz M, Gellert R. The sexuality and quality of life of hemodialyzed patients--ASED multicenter study. J Sex Med. 2009 Apr;6(4):1062-71.
96. Loos C, Briançon S, Frimat L, Hanesse B, Kessler M. Effect of end-stage renal disease on the quality of life of older patients. J Am Geriatr Soc. 2003;51(2):229-33.
97. Malagoni AM, Catizone L, Mandini S, Soffritti S, Manfredini R, et al. Acute and long-term effects of an exercise program for dialysis patients prescribed in hospital and performed at home. Jn, J. 2008 Nov-Dec;21(6):871-8.
98. Malmstrom RK, Roine RP, Heikkila A, Rasanen P, Sintonen H, et al. Cost analysis and health-related quality of life of home and self-care satellite haemodialysis. Nephrol Dial Transplant. 2008 Jun;23(6):1990-6.
99. Manns BJ, Walsh MW, Culleton BF, Hemmelgarn B, Tonelli M, et al. Nocturnal hemodialysis does not improve overall measures of quality of life compared to conventional hemodialysis. Kidney Int. 2009;75(5):542-9.
100. Manns BJ, Johnson JA, Taub K, Mortis G, Ghali WA, et al. Quality of life in patients treated with hemodialysis or peritoneal dialysis: what are the important determinants? Clin Nephrol. 2003 Nov;60(5):341-51.
101. Manns BJ, Johnson JA, Taub K, Mortis G, Ghali WA, et al. Dialysis adequacy and health related quality of life in hemodialysis patients. Asaio J. 2002 Sep-Oct;48(5):565-9.
102. Manu MA, Radulescu S, Harza M, Manu R, Capsa D, et al. Quality of life assessed by SF-36 health survey in renal transplant patients. Transplant Proc. 2001 Feb-Mar;33(1-2):1927-8.
103. Martin CR, Thompson DR. Does dialysis adequacy impact on the quality of life of end-stage renal disease patients?... including commentary by Anthony D with author response. Clinical Effectiveness in Nursing. 2001;5(2):57-65.
104. Martinez-Castelao A, Gorriz JL, Garcia-Lopez F, Lopez-Revuelta K, De Alvaro F, et al. Perceived health-related quality of life and comorbidity in diabetic patients starting dialysis (CALVIDIA study). Jn, J. 2004 Jul-Aug;17(4):544-51.
105. Martins MRI, Cesarino CB. Quality of life in chronic kidney failure patients receiving hemodialysis treatment [Portuguese]. Rev Lat Am Enfermagem. 2005 Sep-Oct;13(5):670-6.
106. Mau L-W, Chiu H-C, Chang P-Y, Hwang S-C, Hwang S-J. Health-related quality of life in Taiwanese dialysis patients: effects of dialysis modality. Kaohsiung J Med Sci. 2008 Sep;24(9):453-60.
107. McFarlane PA, Bayoumi AM, Pierratos A, Redelmeier DA. The quality of life and cost utility of home nocturnal and conventional in-center hemodialysis. Kidney Int. 2003 Sep;64(3):1004-11.
108. McMurray A, Blazey L, Fetherston C. The effect of intradialytic foot pedal exercise on blood pressure phosphate removal efficiency and health related quality of life in haemodialysis patients. Renal Society of Australasia Journal. 2008;4(2):38.
109. Meyer KB, Espindle DM, DeGiacomo JM, Jenuleson CS, Kurtin PS, et al. Monitoring dialysis patients' health status. Am J Kidney Dis. 1994 Aug;24(2):267-79.
110. Merkus MP, Jager KJ, Dekker FW, Boeschoten EW, Stevens P, et al. Quality of life in patients on chronic dialysis: self-assessment 3 months after the start of treatment. The Necosad Study Group. Am J Kidney Dis. 1997 Apr;29(4):584-92.
111. Molsted S, Aadahl M, Schou L, Eidemak I. Self-rated health and employment status in chronic haemodialysis patients. Scand J Urol Nephrol. 2004;38(2):174-8.
112. Molzahn AE, Northcott HC, Hayduk L. Quality of life of patients with end stage renal disease: a structural equation model. Qual Life Res. 1996 Aug;5(4):426-32.
113. Moons P, Vanrenterghem Y, Van Hooff JP, Squifflet J-P, Margodt D, et al. Health-related quality of life and symptom experience in tacrolimus-based regimens after renal transplantation: a multicentre study. Transpl Int. 2003 Sep;16(9):653-64.
114. Muñoz Sancho R, Oto Royo A, Barrio Alonso R, Fernández M. Evolution of the quality of life in patients on haemodialysis: one-year prospective study [Spanish]. Revista de la Sociedad Española de Enfermería Nefrológica. 2006 Jan-Mar;9(1):55-8.
115. Murtagh F. Understanding and improving quality of care for people with conservatively-managed Stage 5 Chronic Kidney Disease – the course of symptoms and other concerns over time: King's College London; 2008.
116. Mustata S, Groeneveld S, Davidson W, Ford G, Kiland K, et al. Effects of exercise training on physical impairment, arterial stiffness and health-related quality of life in patients with chronic kidney disease: a pilot study. International Urology and Nephrology. 2010:1-9.
117. Neipp M, Karavul B, Jackobs S, Meyer zu Vilsendorf A, Richter N, Becker T, et al. Quality of life in adult transplant recipients more than 15 years after kidney transplantation. Transplantation. 2006 Jun 27;81(12):1640-4.
118. Neri L, Rocca Rey LA, Gallieni M, Brancaccio D, Cozzolino M, Colombi A, et al. Occupational stress is associated with impaired work ability and reduced quality of life in patients with chronic kidney failure. Int J Artif Organs. 2009 May;32(5):291-8.
119. Neyra MR, Segura FC, Espejo JLM. Health perceived by patients in CAPD and APD [Spanish]. Revista de la Sociedad Española de Enfermería Nefrológica. 2008;11(2):26-33.
120. Noohi S, Karami GR, Lorgard-Dezfuli-Nejad M, Najafi M, Saadat SH. Are all domains of quality of life poor among elderly kidney recipients? Transplant Proc. 2007 May;39(4):1079-81.
121. Nowicki M, Murlikiewicz K, Jagodzinska M. Pedometers as a means to increase spontaneous physical activity in chronic hemodialysis patients. Jn, J. 2010 May-Jun;23(3):297-305.
122. Oberbauer R, Hutchison B, Eris J, Arias M, Claesson K, et al. Health-related quality-of-life outcomes of sirolimus-treated kidney transplant patients after elimination of cyclosporine A: results of a 2-year randomized clinical trial. Transplantation. 2003 Apr 27;75(8):1277-85.
123. Ogutmen B, Yildirim A, Sever MS, Bozfakioglu S, Ataman R, et al. Health-related quality of life after kidney transplantation in comparison intermittent hemodialysis, peritoneal dialysis, and normal controls. Transplant Proc. 2006 Mar;38(2):419-21.
124. O'Sullivan D, McCarthy G. An exploration of the relationship between fatigue and physical functioning in patients with end stage renal disease receiving haemodialysis. Journal of Nursing & Healthcare of Chronic Illnesses. 2007;16(11c):276-84.
125. Ozminkowski RJ, White AJ, Hassol A, Murphy M. General health of end stage renal disease program beneficiaries. Health Care Financ Rev. 1997;19(1):121-44.
126. Pacheco A, Saffie A, Torres R, Tortella C, Llanos C, et al. Cost/Utility study of peritoneal dialysis and hemodialysis in Chile. Perit Dial Int. 2007 May-Jun;27(3):359-63.
127. Painter PL, Topp KS, Krasnoff JB, Adey D, Strasner A, et al. Health-related fitness and quality of life following steroid withdrawal in renal transplant recipients. Kidney Int. 2003 Jun;63(6):2309-16.
128. Painter P, Carlson L, Carey S, Paul SM, Myll J. Low-functioning hemodialysis patients improve with exercise training. Am J Kidney Dis. 2000;36(3):600-8.
129. Papalois VE, Moss A, Gillingham KJ, Sutherland DE, Matas AJ, et al. Pre-emptive transplants for patients with renal failure: an argument against waiting until dialysis. Transplantation. 2000 Aug 27;70(4):625-31.
130. Parsons TL, Toffelmire EB, King-VanVlack CE. The effect of an exercise program during hemodialysis on dialysis efficacy, blood pressure and quality of life in end-stage renal disease (ESRD) patients. Clin Nephrol. 2004 Apr;61(4):261-74.
131. Peng YS, Chiang CK, Hung KY, Chang CH, Lin CY, et al. Are both psychological and physical dimensions in health-related quality of life associated with mortality in hemodialysis patients: A 7-year Taiwan cohort study. Blood Purif. 2010;30(2):98-105.
132. Perez San Gregorio MA, Martin Rodriguez A, Diaz Dominguez R, Perez Bernal J. Health related quality of life evolution in kidney transplanted patients [Spanish]. Nefrologia. 2007;27(5):619-26.
133. Perneger TV, Leski M, Chopard-Stoermann C, Martin P-Y. Assessment of health status in chronic hemodialysis patients. Jn, J. 2003 Mar-Apr;16(2):252-9.
134. Perlman RL, Finkelstein FO, Liu L, Roys E, Kiser M, et al. Quality of life in chronic kidney disease (CKD): a cross-sectional analysis in the Renal Research Institute-CKD study. Am J Kidney Dis. 2005 Apr;45(4):658-66.
135. Pinson CW, Feurer ID, Payne JL, Wise PE, Shockley S, et al. Health-related quality of life after different types of solid organ transplantation. Ann Surg. 2000 Oct;232(4):597-607.
136. Plantinga LC, Fink NE, Bass EB, Boulware L, Meyer KB, et al. Preferences for Current Health and Their Association With Outcomes in Patients With Kidney Disease. Med Care. 2007 Mar;45(3):230-7.
137. Prasad GVR, Nash MM, Keough-Ryan T, Shapiro RJ. A quality of life comparison in cyclosporine- and tacrolimus-treated renal transplant recipients across Canada. Jn, J. 2010 May-Jun;23(3):274-81.
138. Procaccini DA, Angelini P, Aucella F, Avanzi C, Brusasco S, et al. Health-related quality of life in patients with chronic kidney disease [Italian]. G. 2008 Nov-Dec;25(6):694-701.
139. Pucheu S, Consoli SM, D'Auzac C, Francais P, Issad B. Do health causal attributions and coping strategies act as moderators of quality of life in peritoneal dialysis patients? J Psychosom Res. 2004 Mar;56(3):317-22.
140. Punal Rioboo J, Sanchez-Iriso E, Ruano-Ravina A, Varela Lema ML, Sanchez-Guisande D, et al. Short daily versus conventional hemodialysis quality of life: a cross-sectional multicentric study in Spain. Blood Purif. 2009;28(3):159-64.
141. Rambod M, Bross R, Zitterkoph J, Benner D, Pithia J, et al. Association of Malnutrition-Inflammation Score with quality of life and mortality in hemodialysis patients: a 5-year prospective cohort study. Am J Kidney Dis. 2009 Feb;53(2):298-309.
142. Rathod R, Baig MS, Khandelwal PN, Kulkarni SG, Gade PR, et al. Results of a single blind, randomized, placebo-controlled clinical trial to study the effect of intravenous L-carnitine supplementation on health-related quality of life in Indian patients on maintenance hemodialysis. Indian J Med Sci. 2006 Apr;60(4):143-53.
143. Ravagnani LMB, Domingos NAM, de Oliveira Santos Miyazaki MC. Quality of life and coping strategies in patients undergoing renal transplantation [Portuguese]. Estudos de Psicologia. 2007 May-Aug;12(2):177-84.
144. Rebollo P, Ortega F, Baltar JM, Badia X, Alvarez-Ude F, et al. Health related quality of life (HRQOL) of kidney transplanted patients: variables that influence it. Clin Transplant. 2000 Jun;14(3):199-207.
145. Rebollo P, Ortega F, Baltar JM, Diaz-Corte C, Navascues RA, et al. Health-related quality of life (HRQOL) in end stage renal disease (ESRD) patients over 65 years. Geriatr Nephrol Urol. 1998;8(2):85-94.
146. Reimer J, Franke GH, Philipp T, Heemann U. Quality of life in kidney recipients: comparison of tacrolimus and cyclosporine-microemulsion. Clin Transplant. 2002 Feb;16(1):48-54.
147. Rocco DG, Mercieri A, Yavuzer G. Multidimensional health-status assessment of chronic hemodialysis patients: the impact on quality of life. Eur. 2006;42(2):113-9.
148. Roderick P, Nicholson T, Armitage A, Mehta R, Mullee M, et al. An evaluation of the costs, effectiveness and quality of renal replacement therapy provision in renal satellite units in England and Wales. Health Technol Assess. 2005 Jul;9(24):1-178.
149. Rodriguez AM, San Gregorio M, Dominguez RD, Bernal JP. Differences in health-related quality of life between kidney, heart and liver transplant patients during transplantation process [Spanish]. Psicologia Conductual Revista Internacional de Psicologia Clinica de la Salud. 2008;16(1):103-17.
150. Romao MAF, Romao Junior JE, Belasco AGS, Barbosa DA. Quality of life in patients with chronic renal failure under high-efficiency hemodialysis [Portuguese]. Rev Gaucha Enferm. 2006 Dec;27(4):593-8.
151. Rosas SE, Joffe M, Franklin E, Strom BL, Kotzker W, et al. Association of decreased quality of life and erectile dysfunction in hemodialysis patients. Kidney Int. 2003 Jul;64(1):232-8.
152. Ross EA, Hollen TL, Fitzgerald BM. Observational study of an Arts-in-Medicine Program in an outpatient hemodialysis unit. Am J Kidney Dis. 2006 Mar;47(3):462-8.
153. Russell JD, Beecroft ML, Ludwin D, Churchill DN. The quality of life in renal transplantation--a prospective study. Transplantation. 1992 Oct;54(4):656-60.
154. Saban KL, Bryant FB, Reda DJ, Stroupe KT, Hynes DM. Measurement invariance of the kidney disease and quality of life instrument (KDQOL-SF) across Veterans and non-Veterans. Health and Quality of Life Outcomes. 2010;8.
155. Sandoval-Jurado L, Ceballos-Martinez ZI, Navarrete-Novelo C, Gonzalez-Hernandez F, Hernandez-Colin V. Quality of life in patients with continuous ambulatory peritoneal dialysis [Spanish]. Rev. 2007 Mar-Apr;45(2):105-9.
156. Saracino A, Gollo I, Di Noia I, Caldone MG, Santarsia G, et al. Loss of renal function is associated with deterioration of health-related quality of life in kidney transplant patients. Transplant Proc. 2008 Dec;40(10):3460-5.
157. Santos PR. Correlation between coping style and quality of life among hemodialysis patients from a low-income area in Brazil. Hemodial. 2010;14(3):316-21.
158. Santos PR, Daher EF, Silva GB, Jr., Liborio AB, Kerr LR. Quality of life assessment among haemodialysis patients in a single centre: a 2-year follow-up. Qual Life Res. 2009 Jun;18(5):541-6.
159. Santos PR, Franco Sansigolo Kerr LR. Clinical and laboratory variables associated with quality of life in Brazilian haemodialysis patients: a single-centre study. Rev Med Chil. 2008 Oct;136(10):1264-71.
160. Seica A, Segall L, Verzan C, Vaduva N, Madincea M, et al. Factors affecting the quality of life of haemodialysis patients from Romania: a multicentric study. Nephrol Dial Transplant. 2009 Feb;24(2):626-9.
161. Sennfalt K, Magnusson M, Carlsson P. Comparison of hemodialysis and peritoneal dialysis--a cost-utility analysis. Perit Dial Int. 2002 Jan-Feb;22(1):39-47.
162. Sesso R, Rodrigues-Neto JF, Ferraz MB. Impact of socioeconomic status on the quality of life of ESRD patients. Am J Kidney Dis. 2003 Jan;41(1):186-95.
163. Sesso R, Yoshihiro MM. Time of diagnosis of chronic renal failure and assessment of quality of life in haemodialysis patients. Nephrol Dial Transplant. 1997 Oct;12(10):2111-6.
164. Shield CF, 3rd, McGrath MM, Goss TF. Assessment of health-related quality of life in kidney transplant patients receiving tacrolimus (FK506)-based versus cyclosporine-based immunosuppression. FK506 Kidney Transplant Study Group. Transplantation. 1997 Dec 27;64(12):1738-43.
165. Shrestha A, Basarab-Horwath C, McKane W, Shrestha B, Raftery A. Quality of life following live donor renal transplantation: A single centre experience. Annals of Transplantation. 2010;15(2):5-10.
166. Song YS, Yang HJ, Song ES, Han DC, Moon C, Ku JH. Sexual function and quality of life in Korean women with chronic renal failure on hemodialysis: case-control study. Urology. 2008 Feb;71(2):243-6.
167. Sorensen VR, Mathiesen ER, Watt T, Bjorner JB, Andersen MVN, et al. Diabetic patients treated with dialysis: complications and quality of life. Diabetologia. 2007 Nov;50(11):2254-62.
168. Steiber AL, Davis AT, Spry L, Strong J, Buss ML, et al. Carnitine treatment improved quality-of-life measure in a sample of Midwestern hemodialysis patients. JPEN J Parenter Enteral Nutr. 2006 Jan-Feb;30(1):10-5.
169. Stojanovic M, Ilic S, Stefanovic V. Influence of co-morbidity on health-related quality of life in patients treated with hemodialysis. Int J Artif Organs. 2006 Nov;29(11):1053-61.
170. Sureshkumar KK, Patel BM, Markatos A, Nghiem DD, Marcus RJ. Quality of life after organ transplantation in type 1 diabetics with end-stage renal disease. Clin Transplant. 2006 Jan-Feb;20(1):19-25.
171. Sureshkumar KK, Mubin T, Mikhael N, Kashif MA, Nghiem DD, et al. Assessment of quality of life after simultaneous pancreas-kidney transplantation. Am J Kidney Dis. 2002 Jun;39(6):1300-6.
172. Taji Y, Morimoto T, Fukuhara S, Fukui T, Kuwahara T. Effects of low dialysate calcium concentration on health-related quality of life in hemodialysis patients. Clin Exp Nephrol. 2005 Jun;9(2):153-7.
173. Taji Y, Morimoto T, Okada K, Fukuhara S, Fukui T, et al. Effects of intravenous ascorbic acid on erythropoiesis and quality of life in unselected hemodialysis patients. Jn, J. 2004 Jul-Aug;17(4):537-43.
174. Tajima R, Kondo M, Kai H, Saito C, Okada M, et al. Measurement of health-related quality of life in patients with chronic kidney disease in Japan with EuroQol (EQ-5D). Clinical and Experimental Nephrology. 2010;14(4):340-8.
175. Taskapan H, Ates F, Kaya B, Emul M, Kaya M, et al. Psychiatric disorders and large interdialytic weight gain in patients on chronic haemodialysis. Nephrology. 2005 Feb;10(1):15-20.
176. Thong MSY, van Dijk S, Noordzij M, Boeschoten EW, Krediet RT, et al. Symptom clusters in incident dialysis patients: associations with clinical variables and quality of life. Nephrol Dial Transplant. 2009 Jan;24(1):225-30.
177. Turk S, Atalay H, Altintepe L, Guney I, Okudan N, et al. Treatment with antidepressive drugs improved quality of life in chronic hemodialysis patients. Clin Nephrol. 2006 Feb;65(2):113-8.
178. Turk S, Guney I, Altintepe L, Tonbul Z, Yildiz A, et al. Quality of life in male hemodialysis patients. Role of erectile dysfunction. Nephron. 2004;96(1):c21-7.
179. Unruh M, Benz R, Greene T, Yan G, Beddhu S, et al. Effects of hemodialysis dose and membrane flux on health-related quality of life in the HEMO Study. Kidney Int. 2004 Jul;66(1):355-66.
180. Vasilieva IA. Quality of life in chronic hemodialysis patients in Russia. Hemodial. 2006 Jul;10(3):274-8.
181. Walters BAJ, Hays RD, Spritzer KL, Fridman M, Carter WB. Health-related quality of life, depressive symptoms, anemia, and malnutrition at hemodialysis initiation. Am J Kidney Dis. 2002 Dec;40(6):1185-94.
182. Weng LC, Dai Y-T, Huang H-L, Chiang Y-J. Self-efficacy, self-care behaviours and quality of life of kidney transplant recipients. J Adv Nurs. 2010 Apr;66(4):828-38.
183. White CA, Pilkey RM, Lam M, Holland DC. Pre-dialysis clinic attendance improves quality of life among hemodialysis patients. BMC Nephrol. 2002 Apr 5;3:3.
184. Wight JP, Edwards L, Brazier J, Walters S, Payne JN, et al. The SF36 as an outcome measure of services for end stage renal failure.[see comment]. Qual Health Care. 1998 Dec;7(4):209-21.
185. Wiser NA, Shane JM, McGuigan AT, Memken JA, Olsson PJ. The effects of a group nutrition education program on nutrition knowledge, nutrition status, and quality of life in hemodialysis patients. J Ren Nutr. 1997;7(4):187-93.
186. Wu AW, Fink NE, Marsh-Manzi JVR, Meyer KB, Finkelstein FO, et al. Changes in quality of life during hemodialysis and peritoneal dialysis treatment: generic and disease specific measures. J Am Soc Nephrol. 2004 Mar;15(3):743-53.
187. Yang S-C, Kuo P-W, Wang J-D, Lin M-I, Su S. Development and psychometric properties of the dialysis module of the WHOQOL-BREF Taiwan version. J Formos Med Assoc. 2006 Apr;105(4):299-309.
188. Yildirim A. The importance of patient satisfaction and health-related quality of life after renal transplantation. Transplant Proc. 2006 Nov;38(9):2831-4.
189. Yong DSP, Kwok AOL, Wong DML, Suen MHP, Chen WT, et al. Symptom burden and quality of life in end-stage renal disease: a study of 179 patients on dialysis and palliative care. Palliat Med. 2009 Mar;23(2):111-9.
190. Zimmermann PR, Camey SA, Mari JJ. A cohort study to assess the impact of depression on patients with kidney disease. Int J Psychiatry Med. 2006;36(4):457-68.
